# A Novel Tandem Differential Edge Sensor Layout for Segmented Mirror Telescopes

**DOI:** 10.3390/s23167252

**Published:** 2023-08-18

**Authors:** Yinlong Huo, Fei Yang, Fuguo Wang, Peng Guo, Jiakang Zhu, Yuanguo Liu

**Affiliations:** 1 Changchun Institute of Optics, Fine Mechanics and Physics, Chinese Academy of Sciences, Changchun 130033, China; huoyinlong19@mails.ucas.ac.cn (Y.H.);; 2 University of Chinese Academy of Sciences, Beijing 100039, China

**Keywords:** edge sensor layouts, tandem differential sensor layout, co-phase adjustment, segmented mirror active control

## Abstract

The performance of an active control system, crucial for the co-phase maintenance of segmented mirrors, is closely related to the spatial layout of sensors and actuators. This article compares two types of edge sensor layouts, vertical and horizontal, and proposes a novel tandem differential sensor layout that saves layout space and reduces the number of positioning references. The control performance of this scheme is analyzed in terms of error propagation, mode representation, and the scalable construction of the control matrix. Finally, we constructed a tandem differential-based sensor detection system to examine the performance of edge sensors and the effect of laboratory environmental variables on sensor measurements. Simulations and experiments demonstrate that this scheme has the same ability to fully characterize actuator modification modes as the Keck edge sensor layout. Although the total error multiplier is slightly larger than the latter, it has fewer scalable control matrix types and stronger spatial and segmental shape adaptation capabilities. Actual measurements show that the sensor’s own noise in a tandem differential layout is less than 20 nm, which meets the requirements for future segmented co-phase maintenance. This layout type can potentially be applied to future small and medium-sized segmented splices.

## 1. Introduction

Segmented mirror technology addresses the challenges of manufacturing, transporting, and installing single-piece mirrors, representing a significant trend in the development of large aperture telescopes [1,2,3,4]. During telescope operation, segmented mirrors need to maintain a co-phase state [5]. This state is maintained through an active control system that actively controls the piston, tip, and tilt degrees of freedom of each segment using feedback from relative height measurements between neighboring segments [6,7].

**Figure 1:**
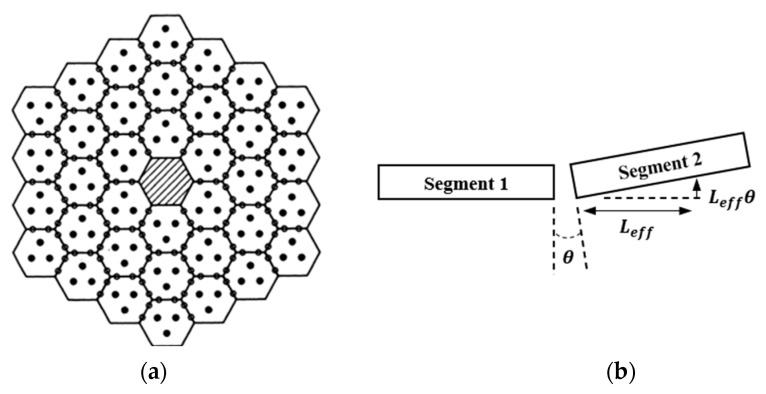
(**a**) Configuration of the primary mirror of Keck. (**b**) Definition of sensor moment arm.

The primary mirror configuration of Keck, illustrated in Figure 1, utilizes edge sensors to measure the relative heights of adjacent segments [8]. However, this method is incapable of detecting the primary mirror’s global motion and is susceptible to time drift and temperature drift. Periodic optical calibration is necessary to correct initial sensor readings. Nevertheless, compared to wavefront detection, this method offers greater accuracy in detecting relative displacements between adjacent segments. The measurement of the edge sensors is sensitive to changes in dihedral angles and relative out-of-plane displacements between segments. The effective measurement arm is the ratio of sensor dihedral angle sensitivity to height sensitivity, denoted as $L_{eff}$. When dihedral sensitivity is zero, there exists a fourth unobservable “focusing mode”, primarily caused by the layout of the sensors. Nonzero dihedral sensitivity makes this mode observable [9].

The layout of edge sensors plays a crucial role in ensuring accurate alignment and co-phasing of the segmented mirror. An effective layout enables highly accurate measurements of segment deformation, facilitating real-time adjustments to maintain optimal optical performance. Table 1 presents the fundamental performance and layout characteristics of various edge sensors employed in segmented mirror telescopes.

**Figure 2:**
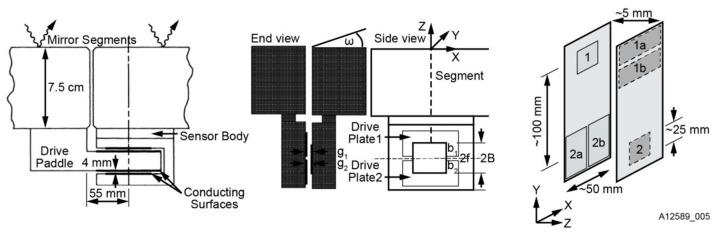
Edge sensor designs for Keck (**left**), TMT (**middle**), and GMT secondary mirror (**right**) [11].

As shown in Figure 2, there are two primary edge sensor layouts: horizontal and vertical. In the horizontal layout, the sensor is positioned parallel to the mirror surface along its edge, while in the vertical layout, it is placed perpendicular to the mirror. A typical example of the vertical layout is the edge sensor used in the TMT [10]. This design employs non-interlocking and non-moving parts to simplify segment replacement. However, such edge sensors generally require customization, resulting in high design and development costs as well as demanding installation precision.

The Keck telescope’s edge sensor layout exemplifies the horizontal layout [8]. The interconnected edge sensors measure the relative height between adjacent segments in a parallel and complementary manner. While this layout offers a smaller error multiplier, it requires more space, higher mounting accuracy, and longer calibration and positioning times. Additionally, this configuration requires more positioning references to ensure the relative position between adjacent sensors and between sensors and sub-mirrors, especially when the sub-mirror shape is non-hexagonal. As a result, this layout may not be suitable for collocating small and medium-sized segmented mirrors.

We propose a novel tandem differential sensor layout that saves layout space and reduces the number of positioning references by mounting two edge sensors in series on the normal of the segmented edge center. This layout also reduces the detection error of rotational motion within the sub-mirror compared to other layouts, making it more versatile and suitable for segmented shapes.

At present, hexagonal shape is the most frequently employed segmentation configuration in the segmentation of primary mirrors [12]. However, recent research indicates that circular segmentation may also be a viable option for building large aperture segmented telescopes. Circular segmentation requires fewer sub-mirror types than hexagonal segmentation, and its support structure design and preparation process are more mature [4]. Furthermore, a study conducted by Cao Haifeng [13] demonstrated that circular and hexagonal segmented primary mirrors with varying apertures exhibit nearly identical modulation transfer functions (MTF), implying the feasibility of circular segmented splices.

This paper conducts a comparative study on the sensor layout of a primary mirror consisting of seven circular segments to ensure the generalizability of the conclusions. Section 2 provides a detailed introduction to the sensor structure layout and the solution of the active control matrix. Section 3 compares the control performance of sensors based on Keck and tandem differential layouts in terms of error propagation, mode representation, and the scalable construction of the control matrix. Section 4 explores the performance of edge sensors in a tandem structural configuration and the influence of laboratory environmental variables on sensor measurements. In the conclusion, this work has been summarized.

## 2. Segmented Active Control Analysis

### 2.1. The Layout of the Edge Sensor Structure

**Figure 3:**
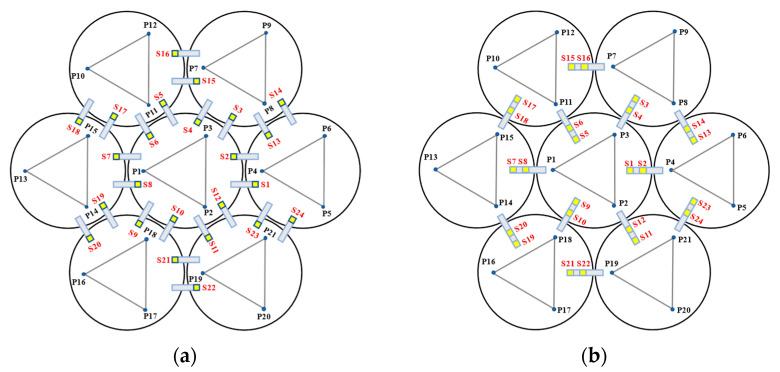
The primary mirror configuration, which displays the positions of actuators and edge sensors, is illustrated with yellow squares (S1 to S24) representing edge sensors and blue dots (P1 to P21) representing actuators. (**a**) describes the edge sensor layout applied to keck, defined as type I; and (**b**) describes the sensor structure layout of the tandem differential, defined as type II.

When Keck’s sensor structure layout [14] is applied to circular segmented mirrors, as shown on the left side of Figure 3, it requires more space, higher mounting accuracy, and longer calibration and positioning times. Additionally, due to the shape difference between circles and hexagons, the positioning process becomes more complex. As a result, this layout may not be suitable for small and medium-sized segmented mirrors.

In order to address the challenges posed by the Keck-type layout, we have developed a new sensor layout scheme, depicted on the right side of Figure 3. In this scheme, the two edge sensors are positioned on the circular centerline of adjacent sub-mirrors. This not only reduces the number of sensor structures but also simplifies calibration positioning compared to the Keck-type layout. Furthermore, this layout offers greater sensitivity to rotational changes within the sub-mirror surface and a smaller measurement error. In practice, considering the structural dimensions of actuators and edge sensors, it is stipulated that actuators and edge sensors should be oriented away from each other. For instance, sensors S1 and S2 should be arranged in a direction away from P4.

**Figure 4:**
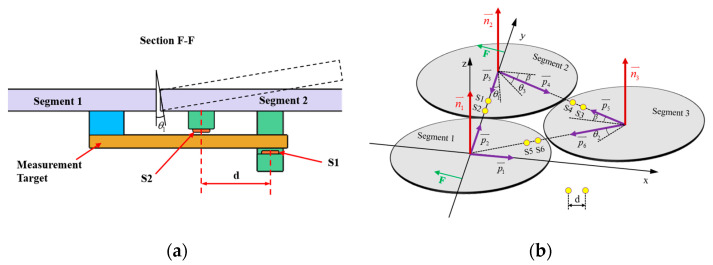
(**a**) Schematic diagram of edge sensor installation and (**b**) orientation definition of the minimum segmentation system. Panel (**a**) is a sectional view of panel (**b**) in the F-F direction. Both sensors, S1 and S2, are mounted on segment mirror 2 with a distance of d between their axes. To enhance the accuracy and reliability of the measurements, differential measurements are utilized to detect the displacement of the target, which is mounted under segment 1.

Figure 4 illustrates a minimal segmentation system that employs the local coordinate system of the fixed segment 1 as the global coordinate system. Vectors $\vec{p_{1}}$ to $\vec{p_{6}}$ are non-collinear within the three segments. Among these vectors, $\vec{p_{1}}$ and $\vec{p_{2}}$ are orthogonal vectors within segment 1, which are conjugate to the local coordinate system. The segmented mirrors can be theoretically viewed as rigid bodies [15], and their orientation vectors can be described as:

(1) $$\left\{\begin{array}{c} \vec{n_{1}}=\frac{\vec{p_{1}}\times \vec{p_{2}}}{\left|\vec{p_{1}}\times \vec{p_{2}}\right|}\\ \vec{n_{2}}=\frac{\vec{p_{3}}\times \vec{p_{4}}}{\left|\vec{p_{3}}\times \vec{p_{4}}\right|}\\ \vec{n_{3}}=\frac{\vec{p_{5}}\times \vec{p_{6}}}{\left|\vec{p_{5}}\times \vec{p_{6}}\right|} \end{array}\right..$$

When the segmented system is disturbed, the change in dihedral angle between segments can be measured by the edge sensor and expressed as:

(2) $$\left\{\begin{array}{c} \theta_{1}=\arcsin(\frac{s_{1}-s_{2}}{d})\\ \theta_{2}=\arcsin(\frac{s_{6}-s_{5}}{d})\\ \theta_{3}=\arcsin(\frac{s_{4}-s_{3}}{d}) \end{array}\right..$$

The vectors $\vec{p_{1}}$ and $\vec{p_{2}}$ remain unchanged because segment 1 is fixed. $R_{x}\left(\theta \right)\;$ is defined as the rotation vector after rotating each segment by an angle of θ around the x-axis of the global coordinate system. Subsequently, the remaining vectors are updated as follows:

(3) $$\left\{\begin{array}{c} {\vec{p_{3}}}^{\prime }=\vec{p_{3}}R_{x}(\theta_{1})\\ {\vec{p_{4}}}^{\prime }=\vec{p_{4}}R_{z}(\beta )R_{y}(\theta_{3})R_{z}(-\beta )\\ {\vec{p_{5}}}^{\prime }=\vec{p_{5}}R_{z}(\beta )R_{y}(\theta_{3})R_{z}(-\beta )\\ {\vec{p_{6}}}^{\prime }=\vec{p_{6}}R_{z}(2\beta )R_{y}(\theta_{2})R_{z}(-2\beta ) \end{array}\right.,$$

where $R_{x}(\theta )=\left[\begin{array}{ccc} 1 & 0 & 0\\ 0 & \cos\theta & -\sin\theta \\ 0 & \sin\theta & \cos\theta \end{array}\right]$, β is the angle between $\vec{p_{4}}$ (or $\vec{p_{5}}$) and the *x*-axis, and is equal to pi/6. The direction vector for each segmented mirror can be calculated by substituting the resulting vector into Equation (2). The components of the direction vector on the *x*-, *y*-, and *z*-axes represent the tip, tilt, and segmented clock, respectively.

### 2.2. Segmented Mirror Active Control

The co-phase state of the segmented primary mirror is preserved by an active control system [16]. This system employs edge sensor data, which measures the relative heights of neighboring segments, to operate actuators to correct the distortions. The three actuators beneath the segmented mirror operate independently. When only one actuator is engaged, the segmented mirror rotates around the line of the remaining two actuators. The geometrical parameters and response coefficients between actuator and sensor for the two layouts are shown in Table 2.

where $C1\;\left(R1\right)\;$ to $C6\;\left(R6\right)$ represent the response coefficients of the sensors and actuators, which were determined using the methods described in the Appendix A. $S_{1}$ to $S_{2}$ represent the variations in readings of adjacent sensors from their ideal values, and $z_{1}$ to $z_{6}$ represent the displacements corresponding to the actuators P1 to P6. The position of the segmented mirror is determined by the arithmetic superposition of the independent movements of three actuators. Therefore, we have the following equation:

(4) $$\begin{array}{l} S_{1}=C_{1}z_{1}+C_{2}z_{2}+C_{3}z_{3}+C_{4}z_{4}+C_{5}z_{5}+C_{6}z_{6}\\ S_{2}=R_{1}z_{1}+R_{2}z_{2}+R_{3}z_{3}+R_{4}z_{4}+R_{5}z_{5}+R_{6}z_{6} \end{array}$$

The correlation between the actuator displacement P and sensor measurement S can be simplified as follows:

(5) S=AP,

where matrix A is the interaction matrix characterizing the response between actuator and sensor. The steps for the construction of the interaction matrix A can be found in the literature [17,18].

### 2.3. Singular Value Decomposition (SVD)

Implementing practical control involves solving the inverse problem of determining the expected length of the actuator based on changes in sensor readings. However, for a superdeterministic system, matrix A is not square, and its inverse does not exist. In general, an exact solution to the system is not possible. Nevertheless, a pseudo-inverse of the response matrix can be created using SVD to obtain an approximate solution to the equation [19]. For the matrix A, the singular value decomposition is as follows:

(6) $$A=USV^{T},$$

(7) $$A^{-1}=VS^{-1}U^{T},$$

where U is an m×n column orthogonal matrix, S is an n×n diagonal matrix whose diagonal elements $s_{i}$ are referred to as the singular values of the matrix A, and V is an n×n  orthogonal matrix with the symbol T for transpose.

Along with computing the inverse, the SVD also provides the eigenmodes (columns of V) of the sensor-actuator system [6]. These eigenmodes represent the shape of the mirror normal modes that respond to sensor readings. This mapping enables the control system to handle individual mirror modes and determine the controllability of the mirror sensing configuration for each mode. The singular values ($s_{i}$) of the control matrix correspond to the controllability of characteristic modes. Smaller singular values indicate that it is more difficult to determine and control the corresponding characteristic mode. A singular value of zero indicates that the corresponding mode is not sensed or controlled by the system. The control matrix analyzed in this study exhibits three zero singularities, which are independent of the sensor configuration. These singularities correspond to eigenmodes related to the rigid body movements (global piston, tip, and tilt) of the entire primary mirror, since such movements do not affect the sensor readings.

## 3. Control Performance of the Sensor Layout Scheme

### 3.1. Determination of Sensor Layout Parameters

The performance of the control matrix is quantified by the minimum singular value except for the unconstrained three modes, global piston, tip, and tilt [20]. Since smaller singular values mean that the corresponding mode is difficult to control, a larger minimum value is required to achieve stable control of the segments. Whereas the singular value of the control matrix depends on the layout of the edge sensors, it is necessary to investigate the relationship between the minimum non-zero singular value and the sensor layout parameters.

For type I sensors, the following constraints apply: (1) no sensor should align with any two actuators within the same segment; and (2) no sensor should interfere with any other sensor within the entire primary mirror configuration. Therefore, the distance (d) and offset (e) between adjacent sensors should satisfy:

(8) $$\left\{\begin{array}{l} a-\frac{\sqrt{3}}{2}d\leq e<a+\frac{\sqrt{3}}{2}d,\;(0\leq d<\frac{m-2a}{\sqrt{3}})\\ a-\frac{\sqrt{3}}{2}d\leq e<\frac{m}{2},\;(\frac{m-2a}{\sqrt{3}}\leq d<\frac{a}{\sqrt{3}})\\ \frac{\sqrt{3}}{2}d\leq e\leq \frac{m}{2},\;(\frac{a}{\sqrt{3}}\leq d\leq r) \end{array}\right..$$

For type II sensors, the distance (d) and offset (e) between adjacent sensors should satisfy: $\frac{a}{2}<e<a,\;0\leq d\leq \frac{m}{2}-e$.

**Figure 5:**
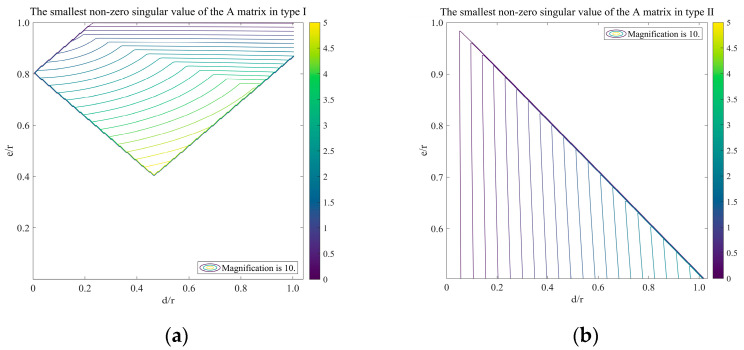
The variation law of minimum non-zero singular value with d, e, and r under two layout states, type I on the left and type II on the right.

Figure 5 illustrates the effect of d, e, and r on the minimum non-zero singular value of the control matrix. The analysis results indicate that:

1. In type I, the minimum non-zero singular value is primarily negatively correlated with e/r and has low sensitivity to variations in d/r. An increase in e represents an increase in the sensor’s measuring arm. However, when e increases to m, the sensor’s sensitivity to the dihedral angle between adjacent sub-mirrors decreases to 0. This results in an undetectable fourth mode (focus mode), consistent with Keck’s analysis;
2. In type II, the minimum non-zero singular value is primarily positively correlated with d/r and is almost insensitive to variations in e/r. An increase in d represents an increase in the sensor’s measuring arm;
3. For the same primary mirror configuration, the maximum value of the smallest non-zero singular value for type I is approximately twice that of type II.

**Figure 6:**
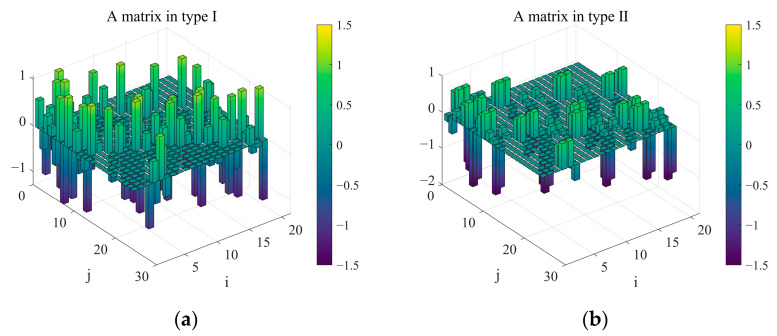
Two types of interaction matrix A, where panel (**a**) describes the value of matrix A in type I, and panel (**b**) is the value of matrix A in type II.

Both types can increase the minimum singular value of the control matrix by increasing the measuring arm of the sensor. However, a longer measuring arm may affect the stability of the relative position between the sensor and the segment. Reasonable e/r, d/r, and a/r ratios were chosen for the Keck telescope to minimize noise multiplication. The exact values of these ratios are not fundamentally meaningful [21]. We determined the parameters of the two layout types based on Keck’s ratios and our analysis, where r = 250 mm, a = 200 mm, m = 505 mm, d1 = 168 mm, e1 = 220.717 mm, d2 = 80.317 mm, and e2 = 150 mm. The parameters d1 (d2) and e1 (e2) represent the distance and offset of adjacent sensors for type I (II), respectively. The values of the A matrix, which were constructed using the method outlined in Section 2.2, are shown in Figure 6.

### 3.2. Comparison of Control Performance between the Two Sensor Layout Types

#### 3.2.1. Error Propagation of the Control System

In segmented active control systems, the quantitative determination of the subsequent response of the actuator can be achieved by the A matrix when random, uncorrelated noise is uniformly injected into all sensors [22].

(9) δp=αδs,

where $\delta_{p}$ and $\delta_{s}$ are the rms values of the actuators and sensors, α is defined as the (overall) noise multiplier. Similarly, we could put random noise into the sensor and determine the rms amplitude $\delta \alpha_{k}$ for each of the 3n−3 modes. Based on the orthogonality of the modes, we have:

(10) $$\delta p^{2}=\sum_{k}\delta p_{k}{}^{2}=\sum_{k}\alpha_{k}{}^{2}\delta s^{2}.$$

It is convenient to arrange the modes in descending order based on their respective error multipliers. Furthermore, in order to evaluate the cumulative effect of the model on the overall error, we define a residual error multiplier $r_{k}$, which includes the error multiplier of the kth mode and all higher modes:

(11) $$r_{k}{}^{2}=\sum_{j\geq k}\alpha_{j}{}^{2}.$$

It is worth noting that the value of $r_{1}$ corresponds to the global error multiplier α. The error multiplier $\alpha_{j}$, which is linked to the *j*th mode, can be demonstrated to be:

(12) $$\alpha_{j}{}^{2}=\sum_{i}{\left(\frac{V_{ij}}{\omega_{j}}\right)}^{2}.$$

**Figure 7:**
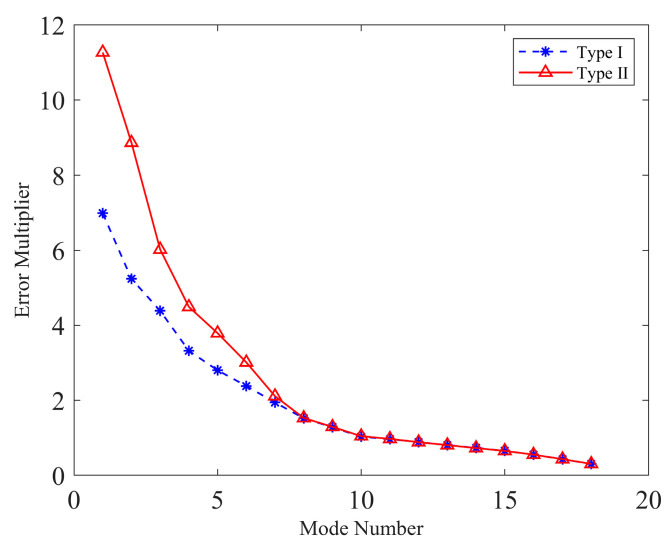
Individual error multipliers for the primary mirror active control systems of telescopes.

Figure 7 illustrates the error multipliers for two different sensor layout types. It can be observed that the error multipliers exhibit a significant decrease as the mode increases. For the last 10 modes, the error multipliers remain virtually identical for both layout types. However, in the first eight modes, type II exhibits higher error multipliers compared to type I, particularly in the first mode. The overall error multipliers are 11.5221 and 17.2904 for type I and type II, respectively.

#### 3.2.2. Characterization of the Actuator Modification Mode

**Figure 8:**
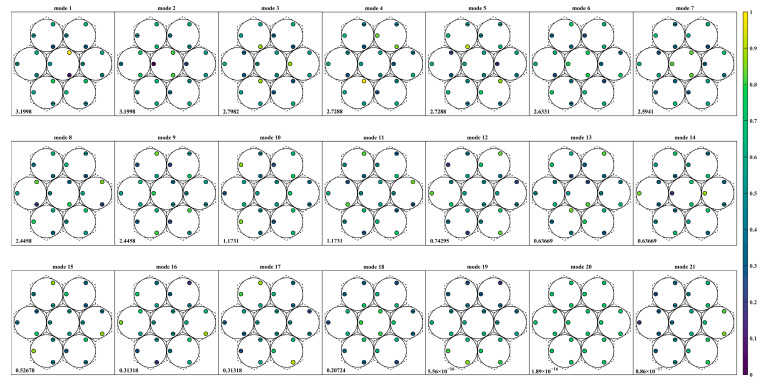
Actuator modification mode based on singular value descent in type I.

**Figure 9:**
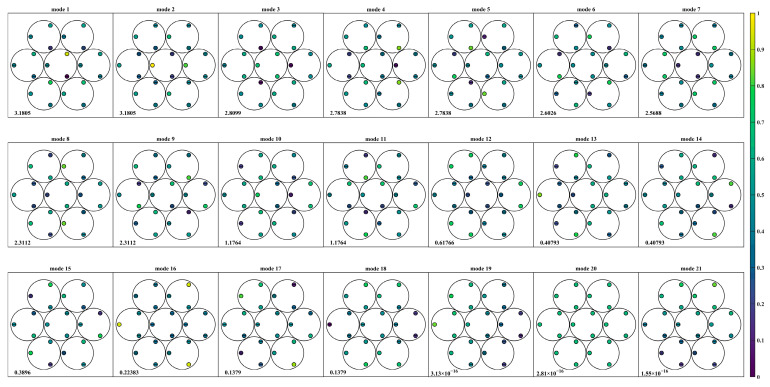
Actuator modification mode based on singular value descent in type II.

The singular value decomposition provides the eigenmodes of the sensor-actuator system. Figure 8 and Figure 9 display the actuator modification modes in descending order of their singular value, with the associated singular value presented at the bottom of each mode. The color of the graph corresponds to the V-matrix column value after global normalization. Additionally, the central horizontal line at the actuator position indicates the negative direction of the actuator.

The comparison of Figure 8 and Figure 9 shows that both sensor layout types can detect 18 modes, except for modes 19 to 21, which correspond to the global mirror motion. Although the actuator modification modes’ order differs between the two types, modes eighteen in type Ⅰ and seven in type Ⅱ represent the focusing mode. This mode is observable due to the non-zero dihedral sensitivity, consistent with the previous analysis. The change in sensor layout approach significantly increases the system’s sensitivity to this mode.

#### 3.2.3. Scalability Analysis of the Control Matrix

In practice, the orientation of triangles formed by actuators may vary from segment to segment [17,23]. However, actuator triangles with the same orientation do not change the fundamental properties of the associated control matrix or affect the derivation of the error multiplier. There will be a slight difference in the construction of the interaction matrix. The complexity of constructing the control matrix is determined by the number of sensor and actuator triangle geometry types. Table 3 summarizes the geometry of a sensor and an actuator triangle below a segment.

The layout types of adjacent sensors with respect to the six actuators are combinations of the types listed in the table. There are eight types of type I and four types of type II. In practice, the actuator triangle must be positioned away from the edge sensor to avoid structural interference, as shown in the left structure of type II in Table 3. Table 4 shows the two commonly used configurations for type II. Type II has fewer sensor and actuator triangle layout types, making it more scalable and reproducible in constructing sensor control matrices. This characteristic provides an advantage in the development of control matrices for future large aperture segmented telescope primary mirrors.

The comparative analysis demonstrates that both schemes have similar total error multipliers and accurately characterize the actuator’s modification mode. However, the scheme proposed in this paper is more compact, with fewer scalable control matrix types and greater spatial adaptability. It also has a lower detection error for rotational motion in the segmentation plane, requires fewer positioning references during installation, and exhibits better segmentation shape adaptability than other layouts. Therefore, the tandem differential edge sensor layout has significant potential for future applications in medium- and small-sized segment splicing.

## 4. Experiment

**Figure 10:**
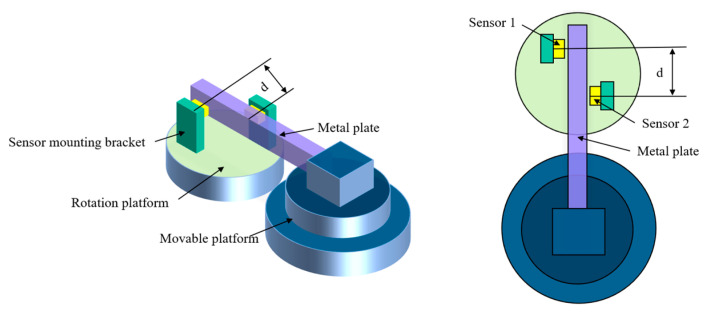
Schematic diagram of the experimental setup.

**Figure 11:**
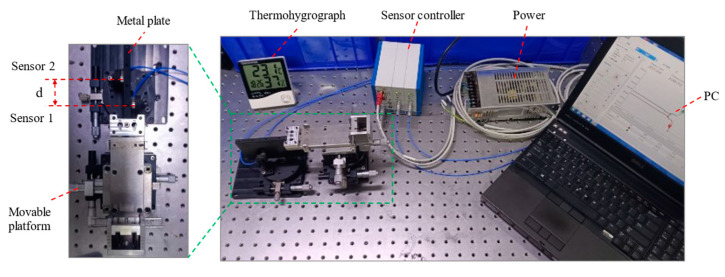
Stability tests performed on edge sensors.

This paper presents an experimental system (see Figure 10 and Figure 11) that evaluates the performance of edge sensors in a tandem layout structure and the impact of laboratory environmental factors on sensor measurements. The system is fixed on an optical platform and uses a DT6230 series capacitive sensor from Micro-Epsilon with a static resolution of less than 5 nm. The sensor is aligned parallel to the test metal plate by adjusting the rotation platform beneath it, and the distance between the metal plate and the two sensors is controlled by a movable platform. A thermohygrometer was used to detect changes during the test. During testing, the center distance between the two sensors is d = 45 mm, and their distance from the test metal plate is approximately 600 μm.

**Figure 12:**
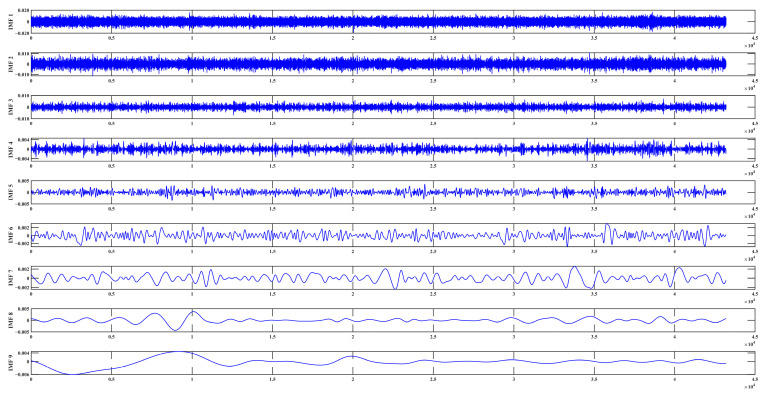
IMF diagram obtained by processing the sensor acquisition signal.

**Figure 13:**
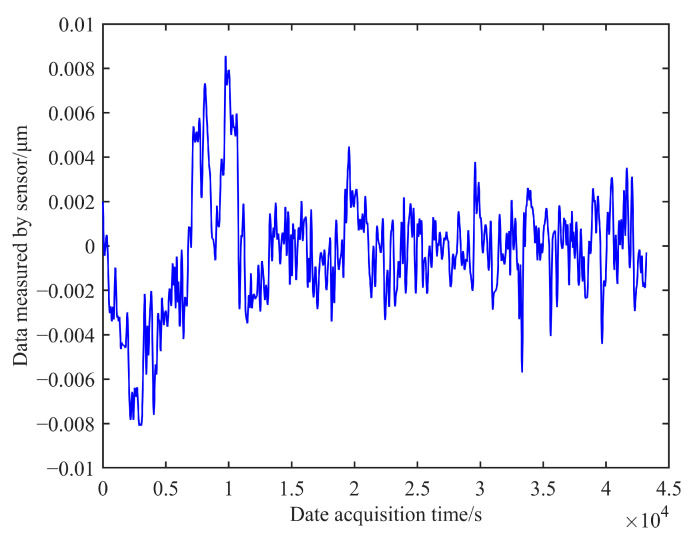
Low-frequency influencing components synthesized in the signal.

The sensor’s performance can be affected by the external environment, with the impact increasing over time. To distinguish environmental noise from the sensor’s inherent noise and to analyze the various frequency components in the acquired signal, this section employs multi-resolution Hilbert–Huang Transform (HHT) technology for signal processing. Taking the data collected by sensor 1 as an example, nine IMF signals are obtained through HHT decomposition, as shown in Figure 12. The amplitude and frequency of each IMF are calculated using the Hilbert Transform, and the low-frequency components are combined. Figure 13 illustrates the synthesized low-frequency signal, which is generated by combining the low-frequency components IMF 5—IMF 9 from the IMF signal.

**Figure 14:**
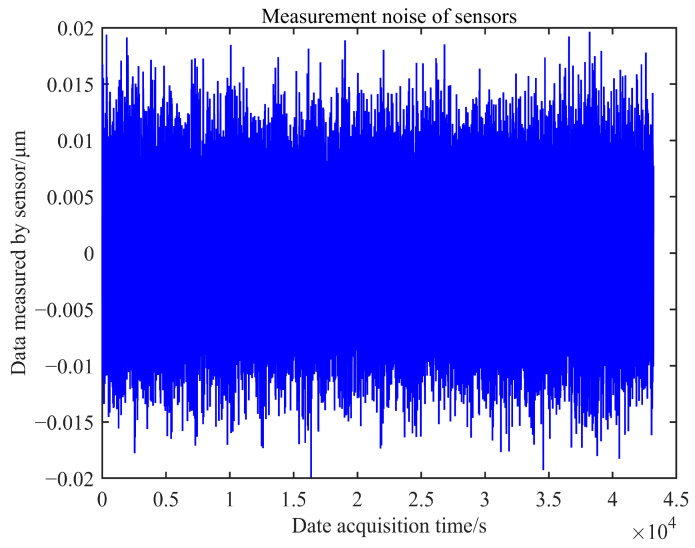
Measurement noise of sensor 1.

The noise background signal containing the measurement noise of the edge sensor can be obtained using the higher-order signal, as shown in Figure 14. The noise signal fluctuates within a range of ±0.02 μm, and the measurement noise error of the edge sensor in a laboratory environment is estimated to be 0.02 μm. Similarly, the measurement noise of sensor 2 in a laboratory environment can be calculated and estimated to be 0.018 μm. These results demonstrate that the sensor’s performance adequately meets the requirements for practical co-phase adjustment.

**Figure 15:**
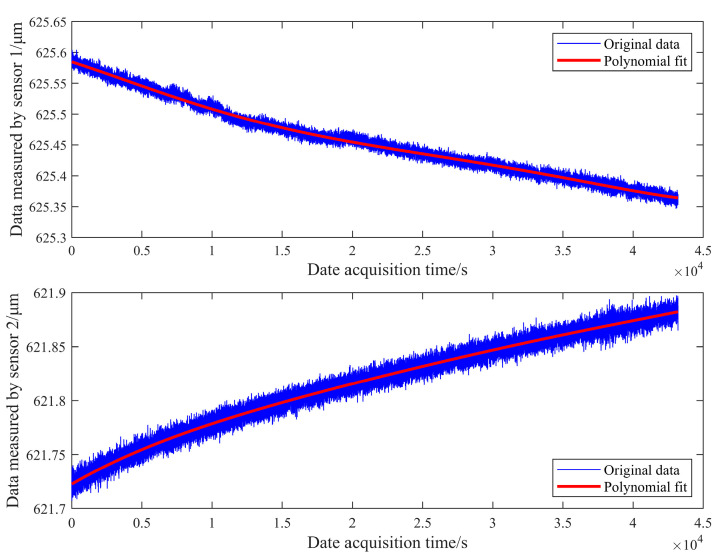
Sensor measurement data in 12 h.

Many telescopes have strict stability requirements. For example, the TMT tertiary mirror system requires its jitter error to be RMS ≤ 69 mas after system calibration and RMS ≤ 7.7 mas after filtering by the adaptive system [24]. Long-term measurements primarily reflect the influence of the environment on sensor measurements. Figure 15 shows the detection of two sensors in a 12 h laboratory environment. The trend of both sensors over time exhibits inverse symmetry: the detection value of sensor 1 gradually decreases while that of sensor 2 increases. This is related to the tandem differential layout of the sensors. The peak-to-valley (PV) values of the fluctuations for the two sensors are 0.2203 μm and 0.1597 μm, respectively.

In the case of a tandem differential layout, the sensor’s own noise must meet the demand for co-phase adjustment. However, disturbances in the laboratory environment can significantly impact the stability of its measurements. Therefore, effective measures must be taken to ensure the measurement environment of the edge sensor during future co-phase adjustments and maintenance.

## 5. Summary and Conclusions

Segmented mirror technology is an important trend in the development of large aperture telescopes. The maintenance of the co-phase state between segmented mirrors is achieved through an active control system, and its performance is closely related to the spatial layout of sensors and actuators. Currently, large, segmented telescopes mainly use vertical and horizontal edge sensor structural layouts. The TMT and Keck telescopes are typical representatives of vertical and horizontal arrangements, respectively. The TMT’s edge sensor design employs non-interlocking and non-moving parts to simplify segment replacement. However, such edge sensors generally require customization, resulting in high design and development costs as well as demanding installation precision. The Keck telescope’s edge sensors measure the relative height between adjacent segments in a parallel and complementary manner, requiring more space and higher mounting accuracy. Furthermore, this configuration requires more positioning references to ensure the installation position of the edge sensor, especially when the segment shape is non-hexagonal. This is unfavorable for the splicing of small-sized segmented mirrors.

We propose a compact differential tandem sensor layout scheme and compare it to the Keck sensor layout in terms of error propagation, mode representation, and control matrix construction. The comparison is carried out on a primary mirror configuration consisting of seven circular segments. Simulation analysis shows that the total error multiplier of the two schemes is not significantly different, and both can fully represent the modification mode of mirror splicing. However, our proposed scheme has a more compact structure and fewer types of scalable control matrices. It requires fewer positioning references during installation and positioning and has stronger spatial and segment shape adaptability. Thus, it can be used as an alternative for future small and medium-sized segment splicing.

In addition, we constructed an experimental system to test the performance of edge sensors under a tandem differential layout and the influence of laboratory environmental variables on sensor measurements. The experimental results show that under a tandem differential layout, the self-noise of the sensor can meet the requirements of co-phase adjustment. During overnight measurements, the PV values of both sensors’ measurements, which varied due to overall environmental disturbances in the laboratory, were 0.2203 μm and 0.1597 μm, respectively.

However, the experiments in this paper focus on analyzing sensor noise measurements in a tandem differential layout without addressing the measurement and calibration of sensor accuracy, stability, and range. These metrics can affect the installation distance between the sensor and the target as well as the final co-phase effect of the system. Future research should carefully consider their potential impact. Additionally, external factors such as time, temperature, and vibration can also affect sensor stability, with varying degrees and mechanisms of influence. This paper only analyzes the effects of the overall laboratory environment on sensor measurements without examining individual sources of error. Future research could use equipment such as vibration isolation platforms and temperature control boxes to study the impact of each source of error separately and propose effective improvement measures. Meanwhile, constructing a segmented mirror test system to test the control performance of the active optical system under a tandem differential layout is also an important area for future research.

## Figures and Tables

**Table 1:** Edge sensor parameters of segmented-mirror telescopes.

	Keck	TMT	E-ELT	LAMOST	SALT	Seimei
Type	Capacitive	Capacitive	Eddy current	Eddy current	Inductive	Inductive
Quantity	168	2952	4924	290	546	72
Range	±12 μm	±500 μm	±200 μm	±1 μm	100 μm	—
Resolution (rms)	<2.5 nm	<5 nm	<5 nm	<1 nm	1 nm	<2 nm
Temperature drift (rms)	3 nm/°C	1 nm/°C	10 nm/°C	6 nm/°C	3.5 nm/°C	—
Time drift	6 nm/week	3 nm/week	10 nm/week	—	10 nm/week	30 nm/night
Layout type	horizontal	vertical	vertical	horizontal	Vertical	horizontal
$L_{eff}$	55 mm	32 mm	≥10 mm	—	—	50 mm

**Table 2:** The geometric parameters and response coefficients between actuator and sensor.

Type I	Type II ^1^
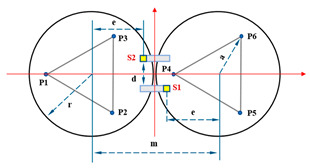	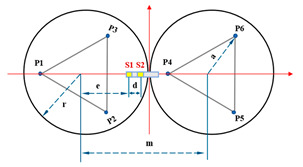
$\begin{array}{l} C_{1}=S_{1}/z_{1}=(2m-a-2e)/3a\\ C_{2}=S_{1}/z_{2}=(2e-2a-\sqrt{3}d-2m)/6a\\ C_{3}=S_{1}/z_{3}=(2e-2a+\sqrt{3}d-2m)/6a\\ C_{4}=S_{1}/z_{4}=(a+2e)/3a\\ C_{5}=S_{1}/z_{5}=(2a-2e+\sqrt{3}d)/6a\\ C_{6}=S_{1}/z_{6}=(2a-2e-\sqrt{3}d)/6a \end{array}$	$\begin{array}{l} C_{1}=S_{1}/z_{1}=(a-2e)/3a\\ C_{2}=S_{1}/z_{2}=(a+e)/3a\\ C_{3}=S_{1}/z_{3}=(a+e)/3a\\ C_{4}=S_{1}/z_{4}=(2e-2m-a)/3a\\ C_{5}=S_{1}/z_{5}=(m-a-e)/3a\\ C_{6}=S_{1}/z_{6}=(m-a-e)/3a \end{array}$
$\begin{array}{l} R_{1}=S_{2}/z_{1}=(a-2e)/3a\\ R_{2}=S_{2}/z_{2}=(2a-\sqrt{3}d+2e)/6a\\ R_{3}=S_{2}/z_{3}=(2a+\sqrt{3}d+2e)/6a\\ R_{4}=S_{2}/z_{4}=(2e-2m-a)/3a\\ R_{5}=S_{2}/z_{5}=(2m-2e-2a+\sqrt{3}d)/6a\\ R_{6}=S_{2}/z_{6}=(2m-2e-2a-\sqrt{3}d)/6a \end{array}$	$\begin{array}{l} R_{1}=S_{2}/z_{1}=(a-2e-2d)/3a\\ R_{2}=S_{2}/z_{2}=(a+e+d)/3a\\ R_{3}=S_{2}/z_{3}=(a+e+d)/3a\\ R_{4}=S_{2}/z_{4}=(2e+2d-a-2m)/3a\\ R_{5}=S_{2}/z_{5}=(m-e-d-a)/3a\\ R_{6}=S_{2}/z_{6}=(m-e-d-a)/3a \end{array}$

^1^ For information on detecting the relative displacement of segmentation mirrors, please refer to the three-mirror minimal segmentation system shown in Figure 4b.

**Table 3:** The geometry of a sensor and an actuator triangle below a segment.

Type I	** 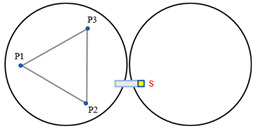 **	** 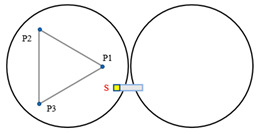 **
	** 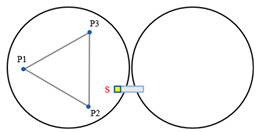 **	** 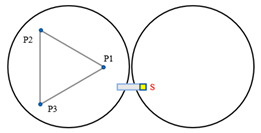 **
Type II	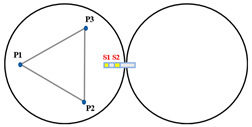	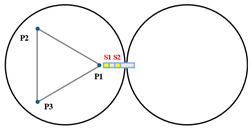

**Table 4:** Common configuration options in type II.

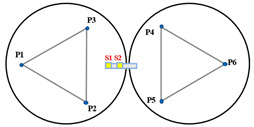	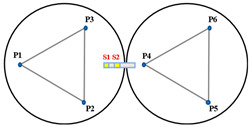

## Data Availability

The data are contained within the article.

## Appendix A

Although the segments are located on a large spherical surface, the relatively small curvature of the primary mirror allows for an accurate description of the sensor–actuator relations using plane geometry. The response coefficients of the sensor to actuator motion can be derived from linear geometric relationships. Taking $R_{1}$ of type I in Table 2 as an example, when only actuator P1 is moved, the left segment will rotate around the line through P2 and P3. The relationship between the displacement $z_{1}$ of actuator P1 and the change in sensor $S_{2}$ can be expressed as follows:

(A1) $$\frac{z_{1}}{\left(\frac{3a}{2}\right)}=-\frac{S_{2}}{\left(e-\frac{a}{2}\right)},$$

then $R_{1}$ is derived from geometric dimensions as follows:

(A2) $$R_{1}=\frac{S_{2}}{z_{1}}=\frac{a-2e}{3a}.$$
